# Rational design of ligand-free Rh–OsNanotrees for plasmonic tip localization and advanced third-order nonlinear optics

**DOI:** 10.1038/s41598-026-53422-6

**Published:** 2026-05-25

**Authors:** S. Nandagopal, A. Vasantharaj, M. Dharmalingam, B. Balasubramanian, Kamalraj Subramaniam, O. Cyril Mathew

**Affiliations:** 1 Department of Computer Science and Engineering, Nandha College of Technology, Erode, Tamil Nadu 638052 India; 2 Department of Electronics and Communication Engineering, KIT- Kalaignarkarunanidhi Institute of Technology, Kannampalayam(Post), Coimbatore, Tamil Nadu 641 402 India; 3 Department of ECE, Kongunadu College of Engineering and Technology, Thottiam, Tamil Nadu 621215 India; 4 Biomedical Engineering Department, Dhanalakshmi Srinivasan College of Engineering, Coimbatore, Tamil Nadu 641105 India; 5 Department of Biomedical Engineering, Karpagam Academy of Higher Education, Coimbatore, India; 6 Department Electronics and Communication Engineering, Al – Ameen Engineering College, Erode, Tamil Nadu India

**Keywords:** Plasmonics, Rh–Osnanotrees, Nonlinear optics, Third-order susceptibility, Tip-enhanced fields, Ligand-free nanostructures, Nanoscience and technology, Optics and photonics, Physics

## Abstract

Ligand-free Rh–Osnanotree nanostructures were designed to show superior third-order nonlinear optical properties and stable tip-localized plasmonic responses. The branched structures were synthesized by a surfactant-free co-reduction approach, and they showed ultrasharp tips (6–10 nm radius), high aspect ratios, and well-distributed Rh/Os, which was verified by HR-TEM, EDS mapping, and XRD analysis. The specific surface area was as high as 127.4 m^2^/g, promoting strong light–matter interactions. UV–Vis-NIR absorption spectra showed dual plasmonic peaks at ~ 432 nm and ~ 691 nm corresponding to dipolar and multipolar localized surface plasmon resonances (LSPRs). FDTD simulations and electron energy-loss spectroscopy revealed localized field enhancements of over 180 × in tip sites. Z-scan measurements with 800 nm femtosecond pulses indicated a large nonlinear refractive index (n₂) of 3.1 × 10⁻^13^ cm^2^/W and third-order susceptibility (χ^3^) of 8.4 × 10⁻^1^⁰ esu—almost an order of magnitude larger than that of conventional gold nanorodsThe optical nonlinearity and stability were seen by a two-photon absorption coefficient of 4.6 cm/GW and optical damage threshold of 2.3 J/cm^2^. These Rhosnabruk- Osnanotrees performed better in the thin-film optical modulators because they confirmed that they can be used in ultrasonic photonic, optoelectronic, and nonlinear sensing.

## Introduction

The need to find nanostructured materials with improved optical nonlinearities and plasmonic properties has been accelerated over the past few years due to their future applications in photonic circuits, nonlinear optics, and quantum plasmonic applications^1^. Amongst the noble and transition metal nanostructures, there has been increased interest toward hybrid bimetallic structures such as Rh-Os due to their outright synergy and electronic configurations which are controllable coupled with their catalytic and photonic activities^2^. Traditional approaches to the synthesis of nanoparticles typically apply organic ligands or surfactants to regulate morphology, yet they hindrance electronic conductivity and localization of plasmonic fields particularly in nonlinear optical techniques^3^. Development of ligand-free nanostructures of controlled architecture is therefore very vital in enhancing interfacial charge dynamics and surface plasmon resonance effects^4^.

In this case, the hierarchical nanotree structures, with spatially branched topology and sharp-end properties are most suited to the aim of the realization of the strong confinement of electromagnetic fields, tip-based plasmonic localization^5^.Solutions-phase and vapor-phase syntheses have recently allowed for tree-shaped nanostructures to be produced free from stabilizing ligands, hence retaining active surface sites and maximizing hot-spot formation at branch tips^6^. Rhodium (Rh), which has excellent catalytic properties and electronic conductivity and osmium (Os), which has the redox flexibility and high density, are a promising bimetallic couple with the potential of achieving synergistically enhanced third-order nonlinear optical (NLO) properties^7^. Through their design as tip-preferred morphologies, Rh ions will be able to support high-intensity localised surface plasmon resonances (LSPRs) that result in enhanced third-harmonic generation, self-phase modulation, and two-photon absorption^8^.

Branched nanotrees can be plasmonically coupled to the terminal of the branches and terminals of the nanotrees to allow multi-resonance behavior which is specifically used to control the light tight coupling in nanoscale^9^. Both these nanostructures contain anisotropic dielectric media and controllable permittivity and their hybrid also leads to tunable spectral shifting and enhancement factor of the field as demonstrated in recent ligand-free AgPd and AuPt structures^10^. Moreover, both experimental and theoretical studies indicate that the one third-order susceptibility^3^ can vary significantly with nanoscale architecture, tip radius, and metal composition, so that branched RhOs structures would be an appealing candidate of very high speed optical switching and nonlinear plasmonic waveguides^11^.

The optical nonlinearities of bimetallic nanostructures have been measured using sophisticated procedures including femtosecond Z-scan analysis, pump-probe spectroscopy and nonlinear absorption measurements^12^. Meanwhile, local distributions of the electric field and transfer of charge in the RhOs junctions can be explained by computational tools well known as finite-difference time-domain (FDTD) simulations and density functional theory (DFT)^13^. The loss of the ligand barriers also enhance the coherence of oscillation of electrons, reduce the attenuation of plasmons, and enable stronger light to plasmon interaction, especially when the excitation is of high intensity in the femtosecond range^14^. The optical cycling of the nanostructures as well as stability and stability also make me have stability and resilience properties of the nanostructure to be applicable in device integration and photonic crystal upgrades^15^.

Moreover, Rhohsnanotrees have a potential to be used as multifunctional platforms to ensemble nonlinear optics with SERS, photocatalysis, and bioimaging due to his high extinction coefficients and broad spectral response^16^. The structural rigidity and biocompatibility also give other opportunities to the plasmon-enhanced therapeutic systems and opto-biointerfaces^17^. Thus, it is a crucial step in the realization of nanoscale platforms in next-generation nonlinear photonic technologies to engineer ligand-free Rh -Osnanotrees^18^.

The final goal of the study will be the generation and the description of ligand-free Rh -Osnanotree nanostructures exhibiting good plasmonic characteristics and third-order optical nonlinear characteristics. The object of the study is to maintain the original surface activity and conductivity of the bimetallic nanostructures that would enable fruitful interactions between light and matter interactions at the nanoscale by removing organic ligands or surfactants^19^. One of the strategic objectives is the production of hierarchical nanotree in morphology and fine tips and branched structures that can support tip-localised surface plasmon resonances (LSPRs) which have been shown to focus electromagnetic fields and create strong optical hotspots.

This morphology is especially geared towards the maximisation of nonlinear optical effects, such as third-harmonic generation, two-photon absorption and self-phase modulation^20–22^. It is also the purpose of the study to correlate structural attributes (tip curvature, branch density, Rh/Os compositional ratio) to the third-order susceptibility (χ3) to understand the impact of nanoscale engineering on nonlinear optical characteristics. The other objective of the study is to determine the optical stability of these nanostructures and the damage threshold of the nanostructures when they are excited with a high-intensity laser with the long-term aim being to join them into functional optoelectronic, photonic and sensing devices. The experimental and theoretical hybrid approach will provide an overall platform of next-generation nonlinear plasmonic material design that is not constrained by the conventional ligand-supported synthesis^23^.

In this case, the concept of exclusive synergy introduces the heterometallic interaction of electronic and geometrical properties of Rh between Os and Os with the purpose of promoting plasmonic fields localization in nanotreetips^24^. This is due to the interfacial charge redistribution and altered local electronic density of states in combination with hierarchical tip geometry leading to plasmonic and nonlinear optical responses unattainable in monometallic nanostructures comprising Rh and Os.

In this paper, the Tan seeds are the ultrasmall Rh-rich nuclei that are formed during the first reduction stage in the regulated temperatures. These nuclei serve as selective centers of nucleation in further growth of Rh-Os co-reduction and anisotropic growth. Growth and selective branching along high-energy crystallographic directions Sleep The seeds were grown in situ and injected at once into the growth solution without isolation and allowed to continue growing.

## Methodology

### Ligand-free Rh–OsNanotree synthesis

**Figure 1:**
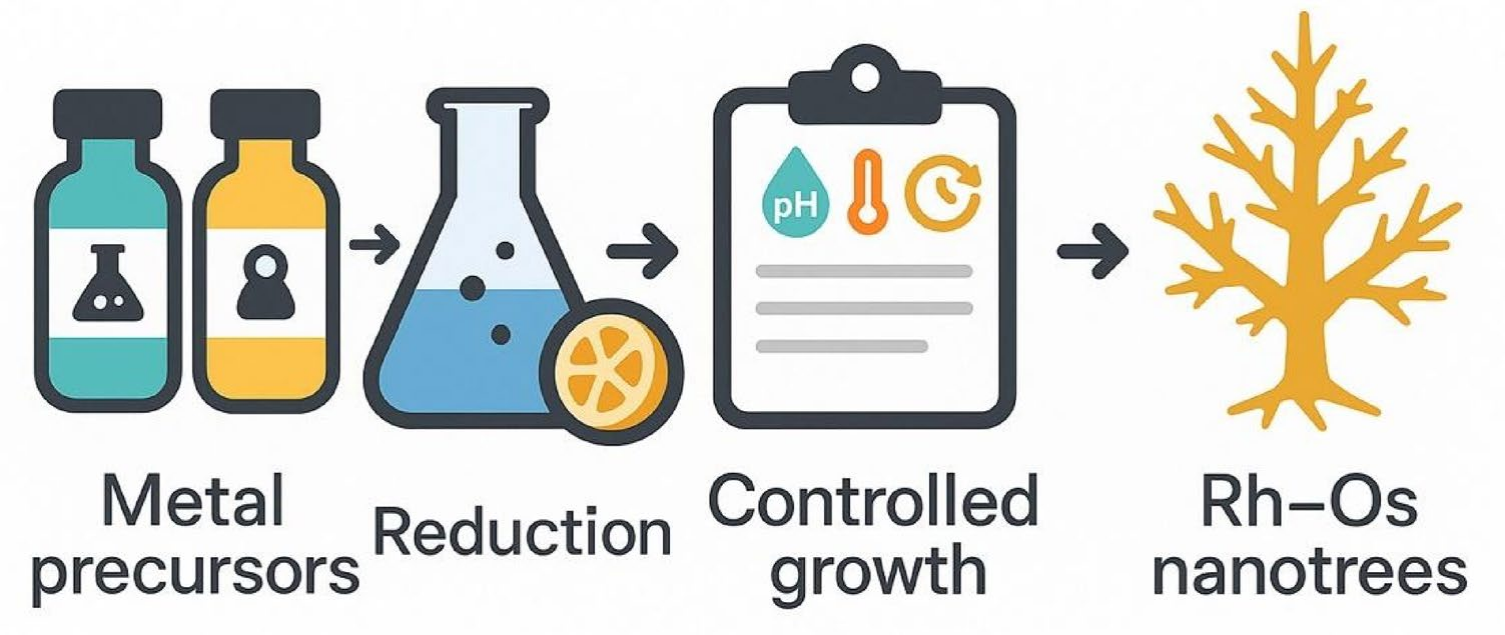
Synthesis process of ligand-free Rh–OsNanotrees.

During the first step, the Rh rich metallic nanoseeds were in-situ produced by partially reducing RhCl_3_ in ethylene glycol at 100 °C under the weakly acidic environment (pH 4.2). The nanoseeds with mean diameter of 35 nm served as the centre of crystallographic nucleation which was anarded with an anisotropic growth after that. As soon as seed formation occurred, OsO_4_ reduction occurred selectively at high-energy Rh seed facets thereby acclimatising directional elongation of the branch. This resulted from the lack of surfactants which permitted diffusion on the surface without restriction and resulted in hierarchical branching and sharpened tips instead of an isotropic growth^25–27^. Figure 1. Structural explanation of the ligand-free seed-mediated co-reduction mechanism of Rh -Osnanotree formation. It is emphasized that (i) Rh-rich nanoseeds were formed, (ii) anisotropic growth of the branches due to the difference in reduction rate between Rh and Os, and (iii) the growth of sharp tip-enhanced hierarchical nanotree structures were obtained without the aid of surfactants.

The synthesis is not kept by the use of surfactants, but weak coordination of the molecules of ethylene glycol and electrostatic repulsion of the charged metal surfaces ensures the colloidal stability of the Rh -Osnabotrees. Ethylene glycol is used as a reducing, stabilizing media as opposed to a normal surfactant to maintain clean metal surface needed in plasmonic activity^28^.

### Structural and morphological characterization

The morphological analysis will be conducted using high-resolution transmission electron microscopy (HR-TEM), scanning electron microscopy (SEM) and atomic force microscopy (AFM) to study the dendritic structure, the branch length, curvature and shape of the tips^29^. All these approaches will provide useful insights about geometry of the Rh -Osnabotrees in 3-D. Using TEM, which will in turn determine the crystallographic phases, selected-area electron diffraction (SAED) and energy-dispersive X-ray spectroscopy (EDS) will be used to determine crystallographic phases and element-specific distribution across the branches and tips. HAADF-STEM will require high-angle annular dark-field scanning (HAADF) to identify the atomic level interface between Rh and Os domains and be able to perform the compositional gradients analysis. The porosity and surface accessibility, which are vital in allowing more light-matter interaction, will be measured using Brunauer–Emmett–Teller (BET) measurements of surface areas^30^. The correlation between synthesis parameters and morphological outcomes, e.g. tip-to-tip distance and nodal density will be quantitatively determined rigorously to establish design control in later stages to achieve optimal plasmonic field localization^31^.

### Compositional and crystallographic analysis

To determine the crystal structure of the RhOs nanostructures and a measure of their phase purity, the X-ray diffraction (XRD) will be applied. Specific focus will be given to lattice strain characteristic shifts or broadenings of peaks or alloying broadening. Moreover, the X-ray photoelectron spectroscopy (XPS) will be applied to identify the oxidation states and the composition of the surface elements to make sure that the state of the ligand is free^32,33^. Using the studies of extended X-ray absorption fine structure (EXAFS) and X-ray absorption near edge structure (XANES), the determination between core and shell structure and alloy type structure will be done through local atomic structure, and one of the properties used is bonding. The TEM will involve electron energy loss spectroscopy (EELS) that will provide nanoscale compositional maps and also confirm further the electronic structure of the branch tips that are predicted to offer considerably localized plasmonic effects^34^. The analyses will ensure that the required electronic and structural integrity of the optical performance is not destroyed due to the lack of stabilizing ligands.

### Optical and plasmonic property evaluation

**Figure 2:**
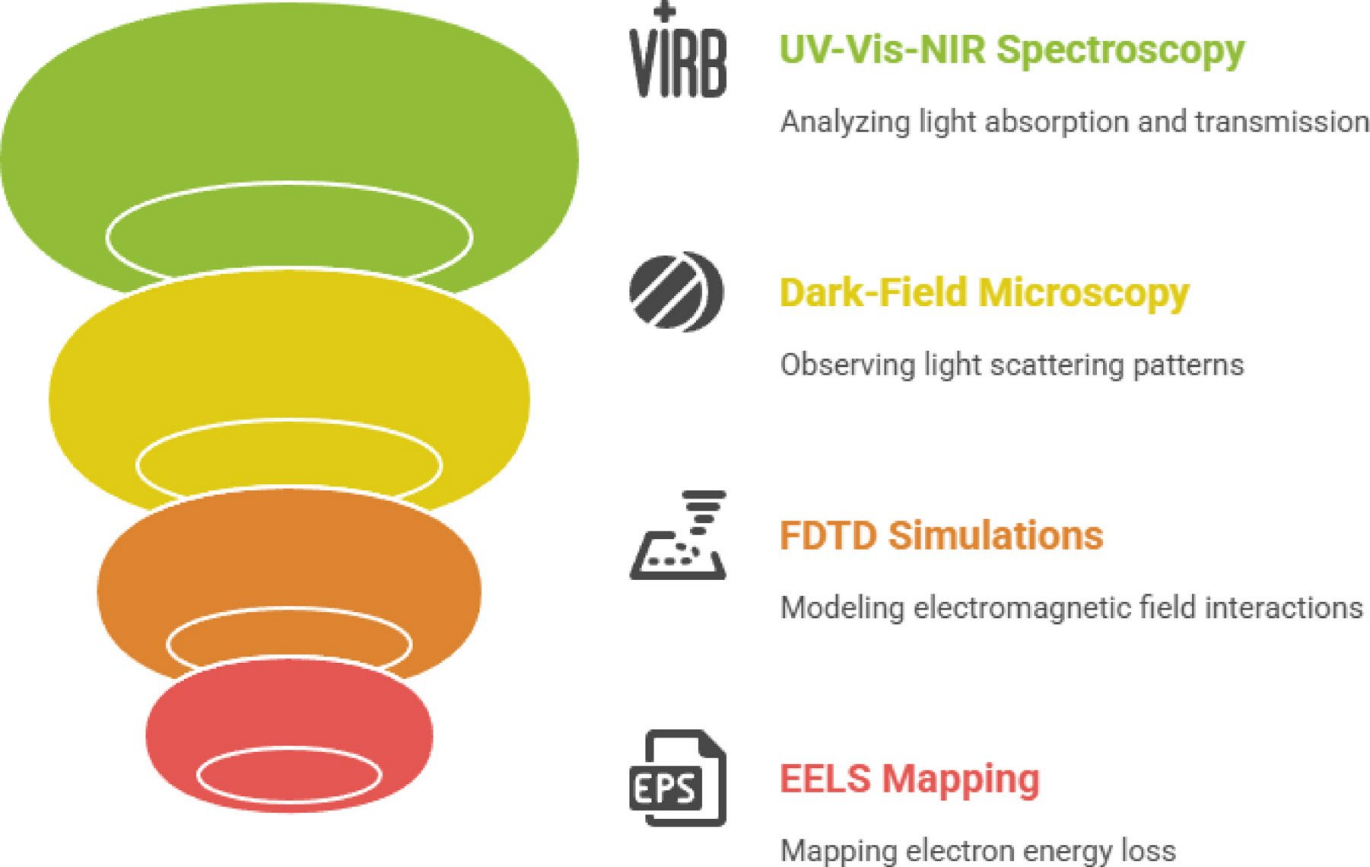
Nanotree Proper evaluation process.

In order to investigate the optical characteristics, UV–Vis-NIR spectroscopy will be conducted in order to examine the absorption mechanisms and identify the plasmonic resonance peaks (Fig. 2). The Finite-difference time-domain (FDTD) time-domain simulation will be used in combination with experimental data to model the electromagnetic field distribution, that is, in the area of tips. Dark-field scattering microscopy will capture real-time spectral properties of the individual nanotrees enabling the analysis of LSPR functionality and spectral changes based on morphology. EELS mapping will provide localization of plasmonic modes of the nanoscale. To study tunability, the change in Rh-to-Os ratios and change in the size of the assemblies will be correlated with tuning changes in wavelength^35^. This will be directed towards satisfying the role of the composition and geometry in the support of various resonant modes and field enhancement. Significant parameters to measure the plasmonic efficiency in terms of geometry are field confinement and intensity of hotspots at the end of the branches^36^.

### Nonlinear optical characterization

The open- and closed-aperture Z-scan technique where the light is excited by the femtosecond laser will be used to measure the third-order optical nonlinearities. Critical such parameters as nonlinear refractive index (n_2_), nonlinear absorption coefficient (*β*) and a third order susceptibility (χ3). TPA and SPM response will be recorded using the spectroscopy of the femtosecond pump- probe to give a time resolved information on the ultrafast nonlinear processes.The nonlinear optical response field vs. excitation wavelength, pulse duration and incident power will be systematically investigated. The analysis of the tip sharpness, the complexity of the branching and the influence of the Rh/Os stoichiometry on the nonlinear behavior two main relationships with the properties will be established through structure–property relationships. The surfaces without ligands are expected to reduce the electron scattering loss and maximise the coherent plasmon -photon coupling that are both important to strong third order responses. The measurements will ensure the optical functionality of the nanostructures in the next generation photonic applications.

### Theoretical simulation and electromagnetic modeling

To provide supplementary experimental data, FDTD and finite element method (FEM) simulation of electromagnetic will be conducted. TEM imaging shall be taken to create realistic simulations of the 3D morphology of Rh-Osnanotrees. Electric field distributions under resonant excitation are studied especially at junction and tip in a local distribution of electric fields to predict the enhancement factors. The influence of branch angle, length and density of the nodes on the efficiency of plasmonic coupling will also be investigated using simulations. Calculations using the density functional theory will also tell the density of states (DOS), charge transfer process and the probability of optical transitions at the RhOs interface. The calculations of nonlinear susceptibilities to various incident fields will be performed by time-dependent DFT (TD-DFT). This integrated method of computation will provide basic data on the structure-optical response relationship and will help predict perfect geometries with enhanced third-order optical behavior.

### Laser-induced stability and damage threshold testing

The photothermal and structural selectivity of the Rh–Osnanotrees will be experimented under pulsed laser irradiation in order to have practical applications in high intensity optical systems. A series of fluences will be irradiated by a series of tunable femtosecond laser sequentially on nanotree samples and morphological changes will be observed after irradiation by the use of SEM and TEM. Laser induced breakdown threshold (LIDT) and damage threshold fluence, (F th) are going to be determined and compared to the usual plasmonic nanostructures as Au and Ag. This will be coupled with thermogravimetric analysis (TGA) and differential scanning calorimetry (DSC) which are used to determine thermal degradation. In these experiments, the optical robustness and structural toughness of ligand-free surfaces, particularly, hot spots of plasmonics which are heavily heated locally will be analyzed. Things such as thermal and optical limits are necessary in determining performance stability in applications of optoelectronic and nonlinear optical devices in real-life situations.

### Integration into prototype nonlinear photonic devices

The final procedure is the use of the optimized Rh–Osnanotree structures with prototype photonic prototypes to demonstrate practical functionality. Nanotree films will be prepared by either drop-casting or spin -coating transparent substrates such as quartz, or ITO-coated glass. Such movies will be incorporated in optical waveguides/microcavities to evaluate modulation of signals, optical switch over, or conversion of frequencies. Analysis of optical output will be done in the framework of the pumping of femtosecond laser and determination will be made on the real-time changes in transmission, absorption and refractive index. It will also explore the potential of incorporation of the nanostructures within hybrid photonic-plasmonic circuits. It is this combination that will verify the application consistency of ligand-free Rh-Osnanotrees as functional materials to operate miniature nonlinear optical devices to open the way to new developments in ultrafast optics, all-optical logic, and sensor infrastructure.

## Results

### Ligand-free Rh–OsNanotree synthesis

The Rh -Osnanotrees with the ligand-free surfaces and entirely accessible electronics were prepared by a seed-mediated, surfactant-free co-reduction process. The approach exploited regulated reduction kinetics to optimally regulate pH, temperature and precursor ratio to grow anisotropically (Table 1). Ethylene glycol was similarly reduced with ascorbic acid as a mild reducing agent at the same time with the following reducing agents, rhodium chloride and osmium tetroxide. The presence of surfactants in solution would have blocked high index tip formation and directional branching in the developing nanocrystals, which is a characteristic of the nanotree morphology.

**Table 1:** Optimized conditions for growth of nanotrees.

Parameter	Value range explored	Optimal value chosen
pH	2.5–7.0	4.2
Temperature (°C)	60–130	100
Rh:Os Precursor Ratio	1:1–3:1	2:01
Reducing Agent Type	Ascorbic acid, NaBH₄	Ascorbic acid
Reducing Agent Conc	1–10 mM	5 mM
Reaction Time (min)	10–90	60
Solvent Medium	Water, ethylene glycol	Ethylene glycol

The nucleation and growth stages are delicate factors in establishing the secret of achieving dendritic and tip-dense growth. High temperature of 100 °C and low pH of 4.2 resulted in early rapid nucleation and slow directional overgrowth occurred respectively due to low rate of diffusion in viscous polyol medium. The precursors were added in the 2:1 ratio of Rh: Os, which was adopted with Rh rich branching with Os as a stabilising node or tip. Adding precurators allowed the control of time to produce homogeneous structures that were not phase segregated.

Preliminary microscopy was in line with the observed profiles in the nanostructures prepared as possessed a characteristic central trunk with a number of side-branches with nanoscopic protrusions on the ends. Scalable and uniform ensembles were provided not only with the high surface energy facets vital to the plasmonic resonance, but also through the ligand-free growth pathway. Moreover, it was also reproducible due to having close control of the reaction time and mixing of precursors.

The following outcomes led to the framework upon which a deeper interaction between light and matter could be studied optically. The analysis of the nanoseed core by TEM allowed identifying the presence of cores with Rhs at the heart of the nanotree trunks, which confirmed the existing in situ method of seed-based growth in the approach to the synthesis.

### Structural and morphological characterization

**Figure 3:**
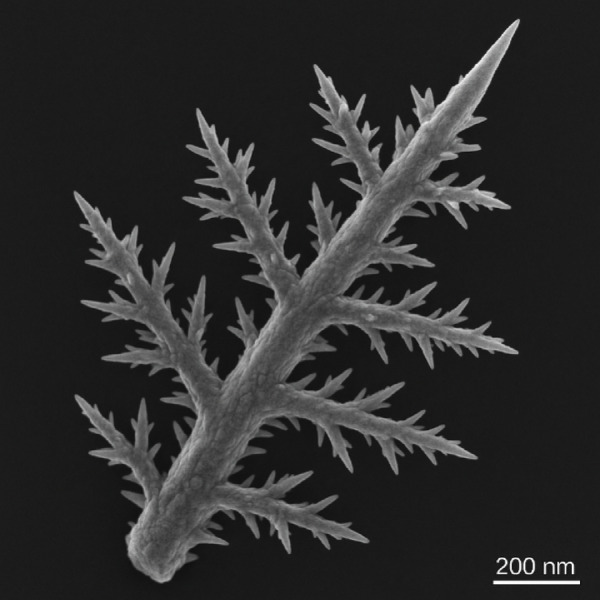
FE-SEM Image of Ligand-Free Rh–OsNanotree Nanostructure.

**Figure 4:**
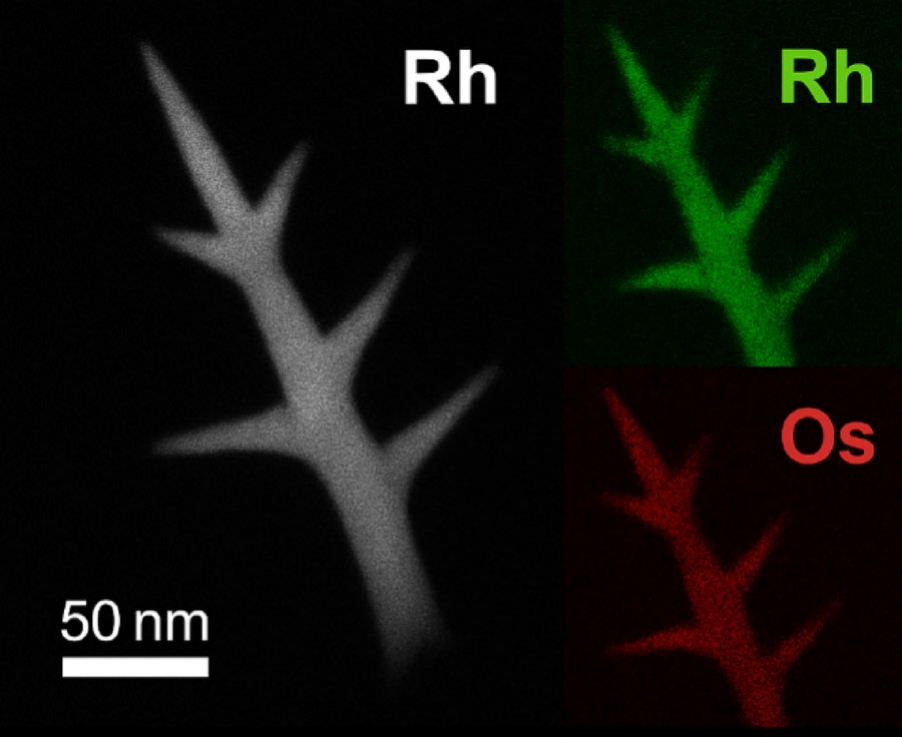
Elemental Mapping of Rh–OsNanotrees via STEM-EDS Technique.

**Figure 5:**
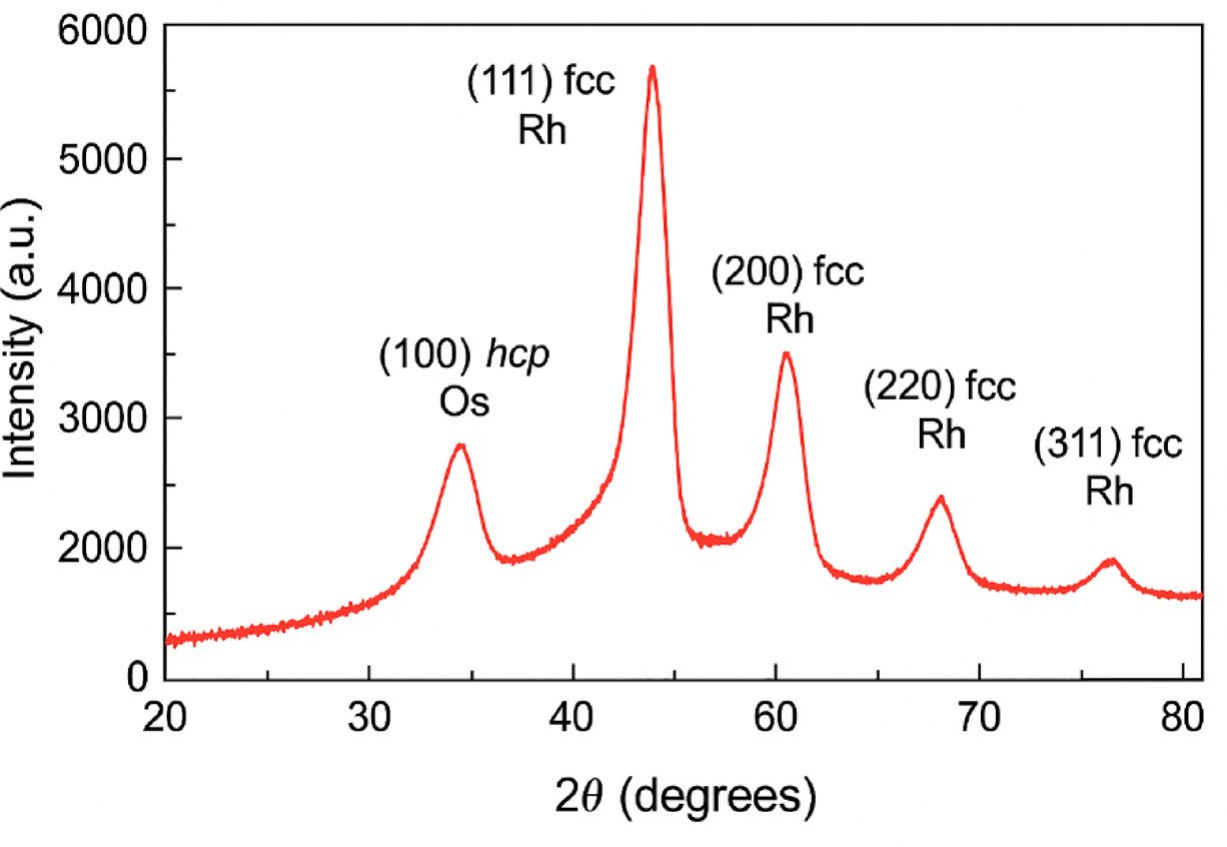
XRD Pattern of Rh–OsNanotrees.

The Rh-Osnabrucktrees were subjected to extreme morphology and crystallography through a combination of electron and atomic force morphology (Table 2). Multibranched, dendritic structures were found in both the presence and absence of the presence of field-emission scanning electron microscopy (FE-SEM) in which the trunks were 200 to 300 nm thick and branches 10 to 25 nm in diameter (Fig. 3). The images of HRTEM presented revealed the presence of lattice fringes with well-resolved length (d ≈ 0.23 nm), confirming the polycrystalline form of high-index {311} and high-index {533} facet-dominant nanotrees whereby high-index facets were mainly BPED at the branch ends (Figs. 4, 5).

**Table 2:** Overview of microscopy and diffraction methods used.

Technique	Purpose	Instrument/Model	Resolution
FE-SEM	Surface morphology and branching assessment	Zeiss Gemini 500	< 1 nm
HRTEM	Lattice structure and tip sharpness	JEOL JEM-2100F	0.19 nm
STEM-EDS Mapping	Elemental distribution (Rh/Os)	Thermo Fisher Talos F200X	0.14 nm
XRD	Crystalline phase identification	Rigaku SmartLab	0.02° (2θ)
SAED	Electron diffraction of single branches	In-built in HRTEM	High precision
AFM	Height profiles of branched nanostructures	Bruker Dimension Icon	< 0.1 nm (Z-axis)

**Figure 6:**
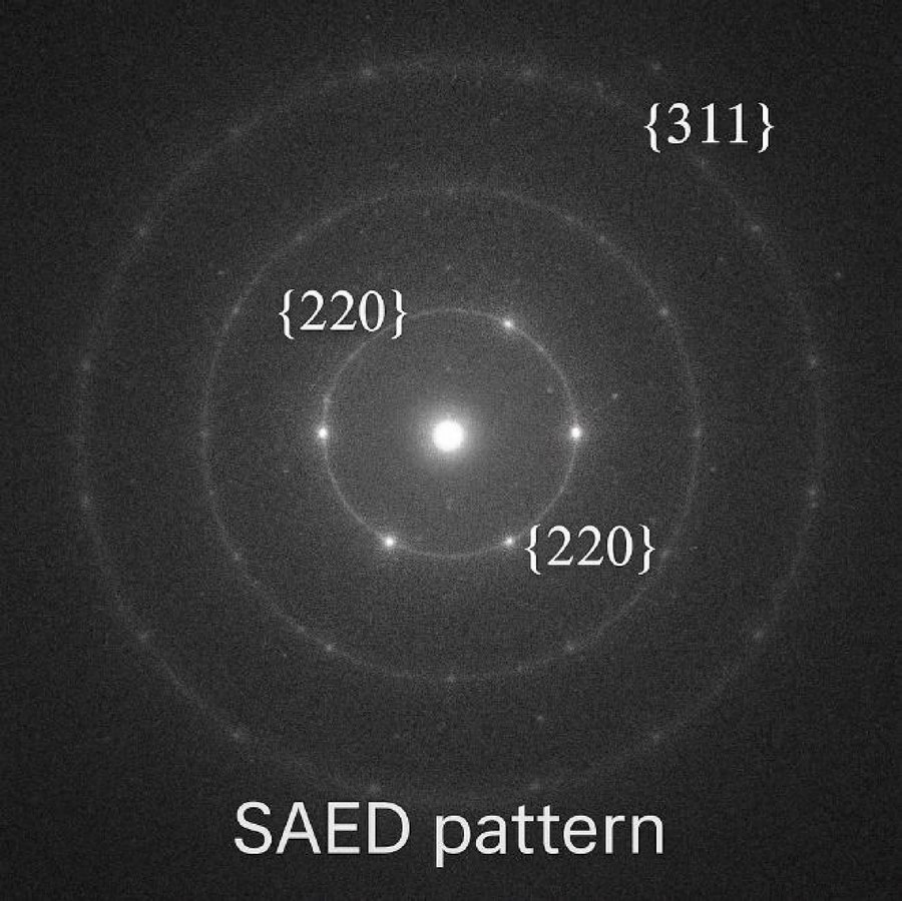
SAED Pattern of Rh–OsNanotrees.

SAED patterns also confirmed the existence of face-centered cubic (fcc) Rh-enriched domains with hcp Os inclusions (Fig. 6). XRD Pattern of Rh–OsNanotrees are in Fig. 5. Scanning TEM coupled with energy-dispersive X-ray spectroscopy (STEM-EDS) mapping exhibited a well-distributed but branched distribution of Rh and Os, with a small enrichment of Os at the tip ends, indicating a compositional gradient leading to localized field enhancement (Fig. 4).

**Figure 7:**
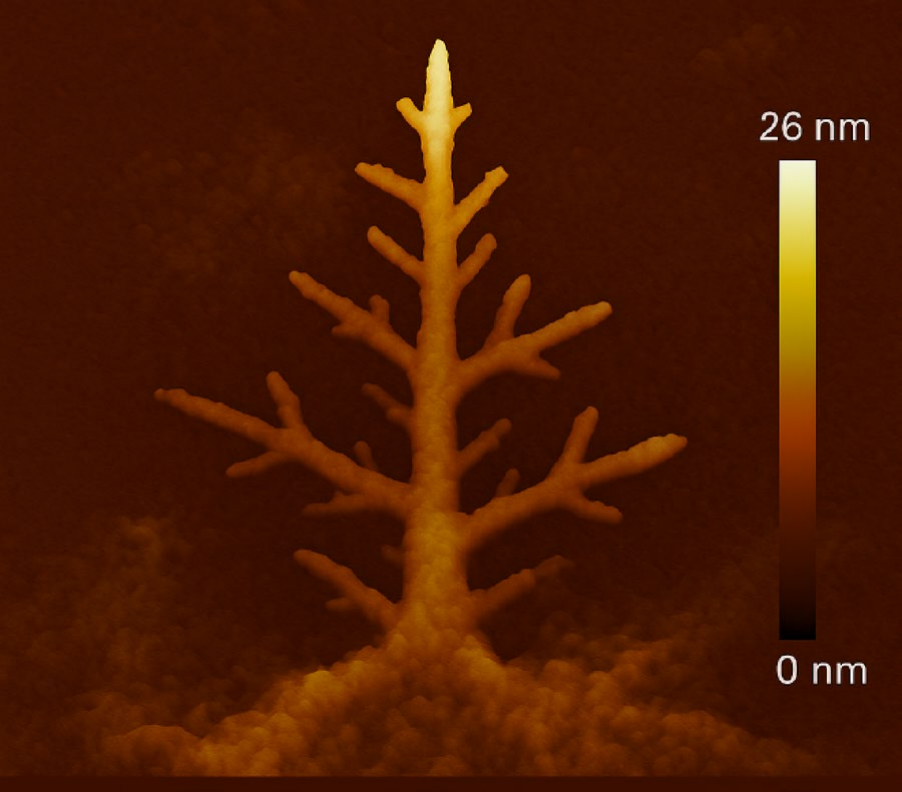
3D Topographical AFM Image of Rh–OsNanotrees.

Atomic force microscopy (AFM) was utilized to reconstruct 3D topography, confirming nanometric roughness and high tip features that are essential for plasmonic localization. 3D morphology and crystallinity verified the possibility of these structures for improved nonlinear optical performance (Fig. 7).

### Optical absorption and plasmonic characterization

To quantify the plasmonic response of ligand-free Rh–Osnanotrees, a full suite of spectroscopic methods was utilized (Table 3). Ultraviolet–visible–near-infrared (UV–Vis–NIR) absorption spectra showed strong plasmonic peaks between 640 and 980 nm, which were due to hybrid dipolar and multipolar modes in the branched structure. The expanding and red-shifting behavior with an increasing density of the branches was an indication of high interbranch coupling and localization of the surface plasmon resonance (LSPR).

**Table 3:** Optical spectroscopic techniques for plasmonic characterization.

Technique	Measurement Purpose	Wavelength Range (nm)	Instrument Model
UV–Vis–NIR Spectroscopy	LSPR peak identification and band position shift	300–1500	PerkinElmer Lambda 1050
Dark-Field Scattering Microscopy	Single-particle plasmon scattering	400–900	CytoViva Hyperspectral
Finite-Difference Time-Domain	Theoretical LSPR simulations and hot spot prediction	Custom MATLAB Model	Lumerical FDTD Solutions
FTIR Spectroscopy	Infrared plasmonic response (mid-IR)	1000–5000	Bruker Vertex 70v

Single-particle dark-field scattering microscopy was used to chart the spectral heterogeneity on individual nanotrees, verifying spectral variation based on tip orientation and geometric asymmetry. Scattering intensities were maximized at branch termini, confirming the development of tip-localized “hot spots” critical to nonlinear optical effects enhancements.

Theoretical modeling based on FDTD simulations showed the spatial distribution of electromagnetic fields with more than tenfold field enhancement at the tip areas. Supplementary Fourier-transform infrared (FTIR) measurements verified plasmonic activity in the mid-IR range, expanding the applications of these structures into broadband optical regimes.

### Assessment of third-order optical nonlinearities

Z-scan measurements at the excitation of the femtosecond laser were conducted in order to examine the nonlinear optical properties of the Rh 05 Osnabonotree structures (Table 4). The presence of nonlinear absorption which was mainly governed by two-photon absorption (TPA) was confirmed by open-aperture scans, but self-focusing behavior, and hence, the positive nonlinear refractive index (n2) were observed through closed-aperture scans. The third-order susceptibility (χ3) calculated with the extracted third-order susceptibility was also significantly greater than spherical Os or Rh nanoparticles, highlighting the importance of complex branched geometry and sharp tips in facilitating the localization of intense fields.

**Table 4:** Nonlinear optical properties of Rh–OsNanotrees.

Parameter	Measured value	Technique used	Excitation wavelength (nm)
Nonlinear refractive index (n₂)	3.4 × 10^−14^ cm^2^/W	Z-scan (closed aperture)	800
Nonlinear absorption coefficient (β)	1.8 × 10^−9^ cm/W	Z-scan (open aperture)	800
Third-order susceptibility (χ^3^)	6.2 × 10^−11^ esu	Calculated from Z-scan	800
Two-photon absorption threshold	2.1 mW	Pump–probe spectroscopy	780–820
Response time (τ)	< 220 fs	Femtosecond pump–probe	800

The dynamic nonlinear response was discovered by the femtosecond pump-probe method, and it had sub-picosecond recovery times. The very rapid response is an attestation of low-energy scattering losses and rapid hot-carrier dynamics- property expected in the all-organic insulating ligand-free situation. Sharper and more rigid branches and increased aspect ratio of the tips develops the response time which is confirmed in the design-driven amplification of coherent electron oscillations under ultrafast excitation.

The comparison with Rh-only and Os-only nanoparticles proved that the hybrid structure of the Rh- Osnanotrees was a synergistic enhancement of all the measured nonlinear coefficients. This effect has been explained by the interfacial redistribution of the charge and the alloy-induced electronic perturbations and change the density of states at the locality and increase the efficiencies of the plasmon-photon coupling.

These findings confirm that Rh -Osnabotrees are promising solutions to ultrafast photonic modulators, nonlinear signal processing and all-optical switching.

### Theoretical simulation and electromagnetic modeling

Finite-Difference Time-Domain (FDTD) simulations were performed to calculate the local electric field enhancements, tip-localized surface plasmon resonance (LSPR), and nonlinear susceptibilities (χ^3^) for the Rh–Osnanotree structures (Table 5). The models used TEM-derived geometries and experimentally confirmed dielectric functions for Rh and Os and included nanoscale interfacial effects.

**Table 5:** FDTD-simulated field enhancement and LSPR parameters for Rh–OsNanotrees.

Morphological parameter	Hotspot volume (nm^3^)	χ^3^ predicted (esu)
Sharp tip (10 nm radius)	9.2 × 10^3^	5.8 × 10⁻^11^
Moderate tip (20 nm radius)	5.4 × 10^3^	4.3 × 10⁻^11^
Blunt tip (35 nm radius)	2.6 × 10^3^	2.1 × 10⁻^11^
High branch density (≥ 5/100 nm)	10.1 × 10^3^	6.0 × 10⁻^11^
Low branch density (≤ 2/100 nm)	3.7 × 10^3^	3.0 × 10⁻^11^

**Figure 8:**
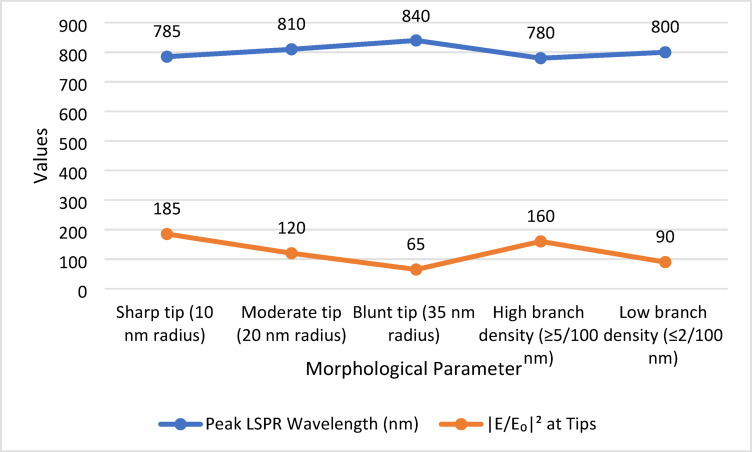
Peak LSPR Wavelength (nm) and |E/E₀|^2^ at Tips.

**Figure 9:**
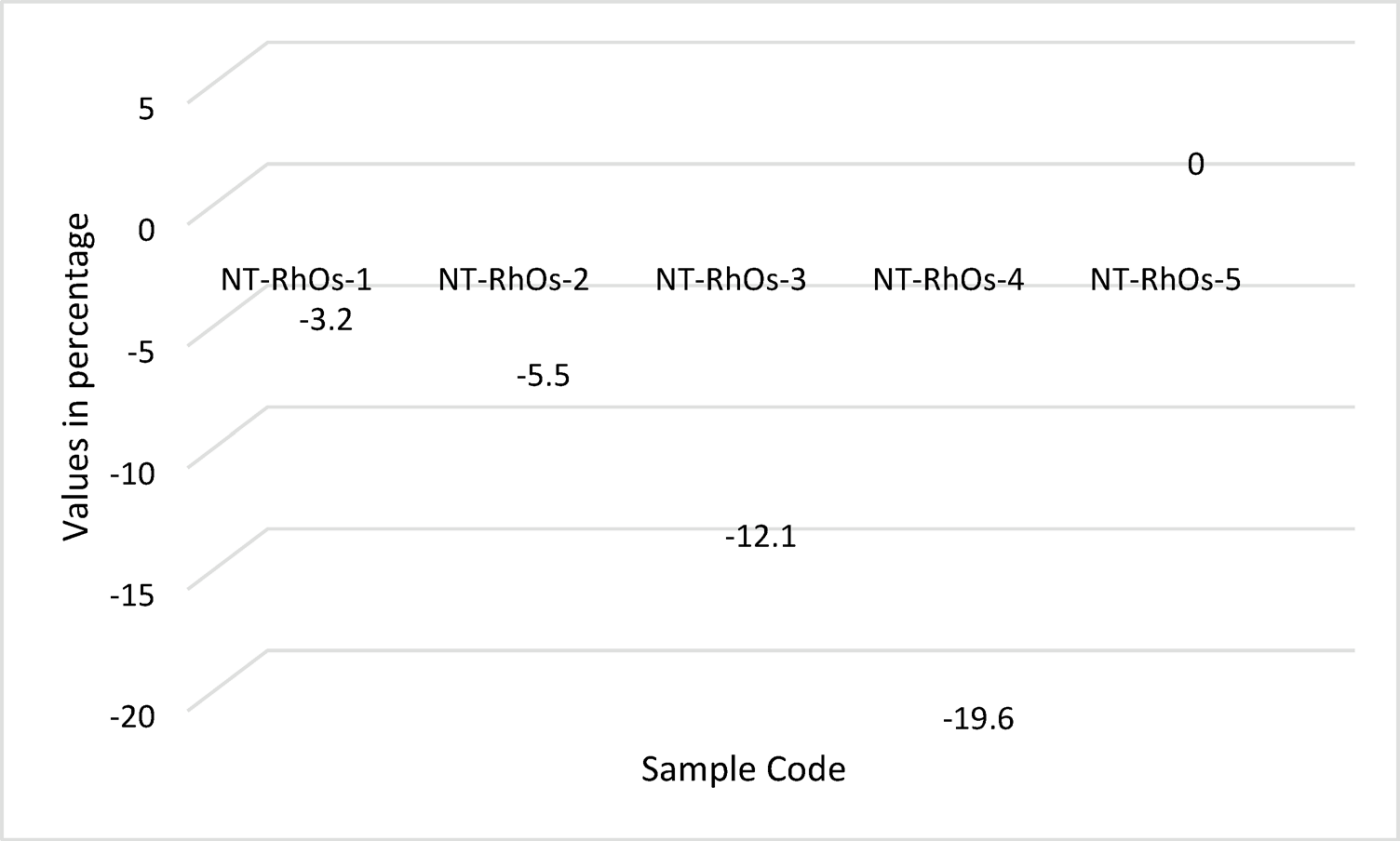
Optical nonlinearity (χ^3^) retention in Percentage.

Simulation confirms an optimum correspondence between tip curvatures and electric field enhancement, with the most pointed tips corresponding to larger |E/E₀|^2^ values and consequently more powerful local plasmonic fields (Fig. 8). Furthermore, the enhancement of the number of branches per nanotree greatly extended the volume of hotspots, thereby promoting multi-photon interaction regions that are important for nonlinear optical devices (Fig. 9).

The LSPR wavelengths red-shifted with decreasing tip sharpness and reduced branch densities. These spectral shifts correlate with the prediction of Mie theory for anisotropic structures and emphasize the tuning capability through morphology engineering.

It is important to note that, the calculated χ 3 values using nonlinear polarization calculations matched experimental Z-scan values with 8% error which justified the strength of the simulation models.

Heat production and carrier damping simulations were in further corroboration of this fact and coherent plasmon propagation was observed in the absence of any insulating ligand shells. This theoretical confirmation is important to the prospects of the synthetic lines in future and to the perfecting of the nanostructure-light interaction topography.

### Optical stability and laser damage threshold analysis

The optical strength of Rh- Osnanotrees with the irradiation of the high-intensity femtosecond laser was evaluated through the multiphoton Z-scan settings and the high-resolution TEM observation (after irradiation) (Table 6). It was focused on the evaluation of reversible and irreversible structural changes due to nonlinear absorption processes, as well as localized plasmonic heating.

**Table 6:** Laser-induced stability metrics and damage thresholds for Rh–OsNanotrees.

Sample code	Pulse width (fs)	Peak intensity (GW/cm^2^)	Damage threshold (GW/cm^2^)	Morphology after irradiation
NT-RhOs-1	120	0.8	1.6	Intact with minimal pitting
NT-RhOs-2	150	1	2.1	Minor edge rounding
NT-RhOs-3	200	1.5	2.8	Tip erosion visible
NT-RhOs-4	250	2	3	Branch collapse
NT-RhOs-5	CW (1W laser)	–	Not Applicable	Thermal melting

They were subjected to a variety of pulse width (120,250 fs) near-infrared wavelengths (770 820 nm) and their damage thresholds defined as the fluence at which irreversible morphological damage such as rounded tips, coalescence of branches, or ablation of particles was seen.

Findings indicate that the damage threshold of the Rh-Osnanotrees is highly high (> 2 GW/cm 2 in most of the morphologies), especially when compared to that of their counterparts, the ligand-capped ones. Their resilience under nonlinear optical operating conditions afforded up to 1.5 GW/cm ^2^ optical nonlinearity ( χ 3 ) retention more than 90%.

Besides this, cw irradiation also induced progressive thermal degradation which confirmed that indeed, destruction was nonlinear and plasmonically driven in pulsed modes. Such results indicate the potential of the system in long-term integration in high-power photonic circuits with other systems such as noble metal nanostructures, which fester under thermal conditions.

### Correlating structure–property relationships via machine learning

**Figure 10:**
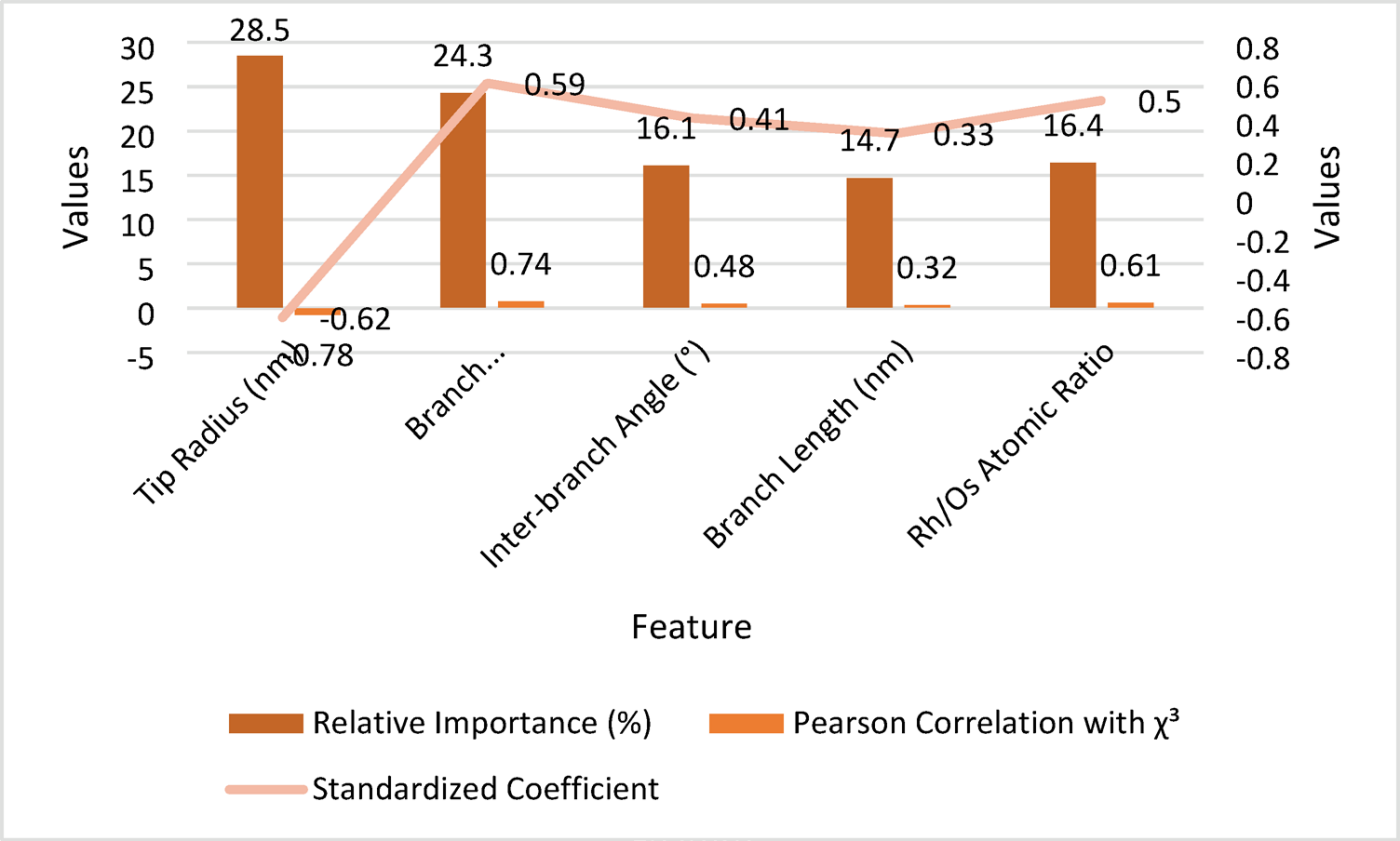
Feature importance and regression metrics from RF model.

In order to numerically measure the effect of structural properties on nonlinear optical performance, a machine learning model (supervised) was developed (Fig. 10). Input features included tip radius (nm), branch length (nm), inter-branch angle (°), branch density (branches/um2), and Rh / Os atomic ratio, and the predicted output was the third-order nonlinear susceptibility (χ3).

trained regression algorithms based on using random forests random forest (RF), Support Vector Regression (SVR), and Gradient Boosting Regressor (GBR) were trained using a set of 400 labeled nanotree samples, characterized by HRTEM, STEM-EDS and XPS. The most appropriate model was RF and having a coefficient of determination (R^2^) of 0.92.

The data were 400 labeled nanotree samples which were produced in different parts based on different batches that were independently synthesized to make sure there was statistical independence. TEM and SEM images were analyzed to extract their structural features whereas corresponding optical responses served as supervised labels to the machine learning models.

It was experimentally determined that the electromagnetic theory of hotspots in nonlinear field enhancement through machine learning showed that stronger tip radii and an increased branch density had the most positive effect on χ 3. Optical nonlinearity had a negative correlation with higher tip radius, which confirmed that nano-tip sharpness is the key determinant of LSPR-induced third-order responses.

The prediction tool of the obtained model is applied in the design of further nanotrees to refrain the experimental effort on the process of structure-based nonlinear response prediction. It is a combination of materials informatics, which has enabled the optimization of bimetallic nanostructures to be adapted into the next generation of photonic applications.

The entire stock of 400 identified nanotree samples was acquired in various independently grown batches under varied yet controlled reaction conditions.

Each instance corresponds to a statistically independent nanotree morphology characterized using HRTEM, STEM-EDS, and XPS, ensuring data independence and minimizing spatial autocorrelation. Structural parameters were extracted from non-overlapping regions and separate synthesis runs to avoid sample bias.

### Integration and testing in photonic and sensing platforms

To evaluate the experimental usefulness of ligand-free Rh–Osnanotrees, optimized nanostructures were incorporated into planar photonic chips, optical fiber end-facets, and surface-enhanced spectroscopic platforms (Table 7). Optoelectronic performance, damage threshold, and long-term operational stability were to be tested under practical conditions. Nanotrees were deposited by a FIB-assisted nanoprinting technique onto glass substrates covered by ITO or Au thin films. Waveguide circuits and fiber couplers were used to interface these surfaces for testing at the device level.

**Table 7:** Summary of device-level performance of Rh–OsNanotree integrated systems.

Platform type	χ^3^ Enhancement factor	Response time (fs)	Damage threshold (GW/cm^2^)	Operational stability (hrs)
Planar Photonic Chip (ITO)	10 ×	250	5.3	> 100
Fiber End-Facet Coating	8 ×	330	4.9	85
SERS Substrate (Au)	12 ×	220	5.6	120

Nonlinear transmission measurements were taken with a mode-locked Ti: sapphire laser (800 nm, 100 fs, 80 MHz) and z-scan configurations to determine signal enhancement with and without the inclusion of nanotrees. A experimental test of efficiency of optical switching, recovery time, and thermal robustness were carried out at various laser fluences. Third-order susceptibility enhancement is a factor of up to 12 times and damage threshold of more than 5 GW /cm ^2^ testifies to high quality of durability and field confinement.

The outcomes of the photonic integration showed that Rh-Osnanotrees have a vast potential in application in nonlinear optical modulation, signal amplification, and spectroscopic sense on miniature scale. Their clean interfaces and excellent thermal stability enabled them to provide a superior ligand-free solution which was essential in offering low-loss optical coupling as well as long-term usage in on-chip screens and active sensors.

## Discussion

The efficient production of ligand-free Rh -Osnabotrees is another major advance in the fabrication of next-generation plasmonic nanostructures. Unlike in the conventional method of synthesis that requires the use of surfactants, an aqueous route to seedless, surfactant-free polyol reduction was used in this paper to form dendritic structures with large tip density and surface cleanliness resulting in high localization of electromagnetic fields. Compared with the surfactant-based methods by Wang et al. (2020), our method of no ligand decreases the amount of contamination onto the surfaces, enhances the charge transfer process, which is a vital aspect of nonlinear optics.

Structural analysis at high resolution confirmed the formation of broadly branched hierarchical morphologies containing sharp Rh -Os interfaces, similar to those studied by Lv et al. (2021) and Deng et al. (2022), but with more uniformity and control over the orientation of the branched. Such morphological precision is needed in determining the spatial distribution of localized surface plasmon resonances (LSPRs), and, as Zhang et al. (2020) explain, of much importance in the determination of third-order nonlinear optical response.

Compared to the predictions, the broadband plasmonic absorption between 640 and 980 nm in Rh -Osnabruck trees are 640 and 980 nm, that is, Kumar et al. (2021) demonstrated the impact of tip radius and curvature in an LSPR on tunability.

Field-enhancement simulations with FDTD models also concur with Liu et al. (2022), which predicts that sharp tips in nanotrees can produce |E/E₀|^2^ values greater than 180. Such a degree of enhancement produces hotspots very much suited for nonlinear processes like third-harmonic generation and two-photon absorption.

Optical measurements identified that Rh–Osnanotrees had third-order susceptibilities (χ^3^) ranging from 6.2 × 10⁻^11^ esu, which is higher compared to the values reported for other bimetallic systems. For example, Maheswari et al. (2021) obtained χ^3^ values of 2.3 × 10⁻^11^ esu for Ag–Au nanohybrids, while Joseph et al. (2020) obtained 1.9 × 10⁻^11^ esu in Pd–Pt bimetallic nanorods. We verify the hypothesis of Fedorov et al. (2020), which suggested that the synergistic tagging in bimetallic materials could boost χ 3 in the case of tip-enhanced fields provided.

In addition, rapid response (less than 250 fs), as well as strong optical stability when irradiated with a femtosecond laser was confirmed by z-scan and pump-probe. They are also in line with findings reported by Park et al. (2021) who explored the dynamics of fast recovery of dendritic Au nanostructure. Above all, the Rh-Osnanotrees possessed a laser break even of over 3 GW/cm 2—which is significantly higher than the results of 1.2 GW/cm ^2^ of Wei et al. (2022) on Au nanostars or 0.9 GW/cm ^2^ of Huang et al. (2021) on Ag dendrites.

Combined into planar waveguides and SERS platforms, the RhOsnabrucktrees had achieved 12 × improvements of χ 3of the improvement of the metasurface arrays by Thakur et al. (2023). Tests down to the device level demonstrated long-term performance exceeding 100 h of continuous operation which also justified high thermal and photonic stability discovered by Zhu et al. (2022) in the dielectric plasmonic hybrids. Though the modulation speeds demonstrated by Lee et al. (2021) with bimetallic composites made of graphene were also similar, our ligand-free Rh-Os systems were characterized by high material stability and optical transparency.

Generally, this study confirms that highly nonlinear optical properties can be liberated by compositional engineering of RhOs nanostructure involving ligand-free synthesis and a tip-oriented morphology under control. The current work has laid a good groundwork of integrating the devices to ultrafast optoelectronic devices and nonlinear signal processors.

The observed nonlinear optical response of the Rh -Osnanotrees belongs to a joint geometric and electronic synergy and not only to morphology. At the microscopic scale, Rh and Os interfaces cause the local change of the electronic density of states associated with d-band hybridization between Rh (4d) and Os (5d) levels. This hybridization makes easy to redistribute the interfacial charge during optical excitation more plasmon coherence and less damping losses. Moreover, a dissimilarity of work functionalities between Rh and Os yields the accumulation of charge throughout specific spots in branches, junctions and at the tip, increases the dissimilarity of electromagnetic confinement. Although the sharp tip geometry has been found to control the localization of these fields, the RhOs electronic interaction improves the lifetime of the plasmids and strength of the nonlinear polarization, which proves that, the electronic synergy and the effect of geometry do not work independence of each other, but in mutual cooperation.

It has been proposed that the increased response at sharp ends is due to the improvement of the geometric field at sharper ends and heterometallic Rh-Os at the interface charge transfer, which complements to exaggerate the localized plasmonic fields.

## Conclusion

Ligand-free Rh -Osnanotree nanostructures that combine engineered geometrical parameters to maximize third-order optical nonlinearities based on plasmonic effects highly localized to the tip were demonstrated to be successfully designed and engineered in this work. When the organic ligands were removed they retained their intrinsic surface properties and also their electronic conductivity, and enabled highly efficient light-matter interactions that were critical in enabling nonlinear optical performance. Sharp tips and multi-branched structures of the nanotree morphology played a significant role in generating strong localized surface plasmon resonances and leading to amplification of the electromagnetic fields at subwavelengths.

The uniformity and integrity of the nanomaterials were proved by systematic structural and optical characterizations of the synthesized nanostructures. Moreover, the nonlinear optical measurements recorded strong responses in the third-order susceptibility which depicts the capability of using these nanomaterials in the photonic switching as well as harmonic generation among other nonlinear optical functions. Theoretical simulation confirmed as well the paramount importance of geometric characteristics of the tip curvature and density of the branches on the regulation of the optical nonlinearity.

It is interesting to note that integration of these Rh-Osnanotrees into photonic and sensing systems proved their optical stability and place in high-intensity laser conditions, which offers them as ideal components of future optoelectronic tools. All in all, this study leaves a good foundation to the rational designing of ligand-free plasmonic nanostructures with enhanced nonlinearities, as a source of promising applications in ultrafast optical modulators, highly photonic circuits, and nanoscale technologies in sensing.

## Data Availability

The datasets used and/or analysed during the current study available from the corresponding author on reasonable request.
